# Modulation of Gut Microbiota of Overweight Mice by Agavins and Their Association with Body Weight Loss

**DOI:** 10.3390/nu9090821

**Published:** 2017-08-23

**Authors:** Alicia Huazano-García, Hakdong Shin, Mercedes G. López

**Affiliations:** 1 Departamento de Biotecnología y Bioquímica, Centro de Investigación y de Estudios Avanzados del IPN, Unidad Irapuato 36821, México; ahuazano@ira.cinvestav.mx; 2 Faculty of Food Science and Biotechnology, College of Life Science, Sejong University, Seoul 05006, Korea; hakdong.shin@gmail.com

**Keywords:** agavins, prebiotics, microbiota, overweight, body weight loss, short chain fatty acids

## Abstract

Agavins consumption has led to accelerated body weight loss in mice. We investigated the changes on cecal microbiota and short-chain fatty acids (SCFA) associated with body weight loss in overweight mice. Firstly, mice were fed with standard (ST5) or high-fat (HF5) diet for five weeks. Secondly, overweight mice were shifted to standard diet alone (HF-ST10) or supplemented with agavins (HF-ST + A10) or oligofructose (HF-ST + O10), for five more weeks. Cecal contents were collected before and after supplementation to determine microbiota and SCFA concentrations. At the end of first phase, HF5 mice showed a significant increase of body weight, which was associated with reduction of cecal microbiota diversity (PD whole tree; non-parametric *t* test, *p* < 0.05), increased Firmicutes/Bacteroidetes ratio and reduced SCFA concentrations (*t* test, *p* < 0.05). After diet shifting, HF-ST10 normalized its microbiota, increased its diversity, and SCFA levels, whereas agavins (HF-ST + A10) or oligofructose (HF-ST + O10) led to partial microbiota restoration, with normalization of the Firmicutes/Bacteroides ratio, as well as higher SCFA levels (*p* < 0.1). Moreover, agavins noticeably enriched *Klebsiella* and *Citrobacter* (LDA > 3.0); this enrichment has not been reported previously under a prebiotic treatment. In conclusion, agavins or oligofructose modulated cecal microbiota composition, reduced the extent of diversity, and increased SCFA. Furthermore, identification of bacteria enriched by agavins opens opportunities to explore new probiotics.

## 1. Introduction

Agavins are branched neo-fructans found in *Agave* plants, which contain a mixture of β(2-1) and β(2-6) linkages [1,2]. The degree of polymerization (DP) and the chemical structure of agavins become more complex as the plant ages. Plants from two to four years old have a high content of agavins with low DP and simpler chemical structures, while plants from five to seven years old contain a large proportion of high-DP agavins and highly-complex chemical structures [3].

Agavins act as prebiotics inducing benefits to host health by providing specific changes in the composition and/or activity of the gut microbiota [4]. Due to their structural complexity, endogenous gastrointestinal enzymes cannot degrade agavins during their passage through the stomach and the small intestine; so they reach both the cecum and colon, where they are fermented by saccharolytic microbiota present in these sites, producing short chain fatty acids (SCFA), mostly acetate, propionate, and butyrate. SCFA are very important because they reduce body weight gain, through G-protein-coupled receptors (GPRs), influencing the secretion of hormones involved in appetite control [5,6,7]. In addition, SCFA increment through agavins fermentation in both cecum and gut induces a pH drop; which might change the intestinal microbiota structure [8,9].

On the other hand, earlier investigations showed that mice fed with standard or high-fat diets with agavins of low DP led to body weight loss [10,11,12]. However, the microbial mechanisms remain unclear [12,13]. New molecular techniques that enable analysis of non-cultivable bacteria are starting to be applied in studies investigating the impact of prebiotics on the cecal microbiota. For example, investigations examined the effects of oligofructose (linear fructans) on cecal microbiota using the 16S rRNA gene sequencing technique, showed that the intake of oligofructose in mice not only stimulated the growth of *bifidobacteria* and *lactobacilli*, but also increased other bacteria such as *Streptococcus*, *Clostridium*, *Enterococcus*, *Olsenella*, *Akkermansia*, and *Allobaculum* [14,15]. The abundance of specific taxa, such as *Bifidobacterium* spp. and *Akkermansia muciniphila* has been negatively associated with inflammation in adipose tissue, circulating glucose, leptin, triglycerides, and insulin [16], whereas the enrichment of *Allobaculum* has been associated with body weight loss in obese mice [17]. On the other hand, Firmicutes and Bacteroidetes are usually the most abundant members of the cecal microbiota; however, the ratio of these bacterial groups can change over time or by different factors, such as age, environment, or diet, and especially those with a high fat content [18,19,20].

In the present work we used agavins from four-year-old *Agave tequilana* plants containing a high proportion of short DP fructans, and studied the response on the microbiota of mice, continuing our previous study on prebiotic supplementation in overweight mice [21]. Here we present changes of cecal microbiota after a diet shift and agavins supplementation, and the possibility of their association with body weight loss in overweight mice. Our hypothesis was that agavins supplementation might improve the host health, through the enrichment of probiotic bacteria, in relation to the diet shift alone.

To our knowledge, this is the first report on the global effects of a diet shift and agavins supplementation on the cecal microbiota composition through a 16S rRNA analysis in overweight mice. Finally, agavins (branched fructans) effects were compared to oligofructose (linear fructans), which was used as a positive control to evaluate the cecal microbiota changes.

## 2. Materials and Methods

### 2.1. Animals and Diets

Forty-two male C57BL/6 mice (12 weeks old at the beginning of the experiment were obtained from the Universidad Autonoma Metropolitana, Mexico City, Mexico) and housed in a temperature and humidity controlled room with a 12 h light-dark cycles. Mice were maintained in individual cages since water intake containing the fructans was measured every day. The animals were subject to a two-phase trial, the first to gain weight and the second to lose weight (Supplementary Figure S1). In the first phase mice were fed with standard (*n* = 12; 5053 Lab Diet, St. Louis, MO, USA) or high-fat diets (*n* = 30; 58Y1 Test Diet, St. Louis, MO, USA) for five weeks. The standard diet (5053 Lab Diet) contained 62.4% calories from carbohydrates (28.6% starch, 3.24% sucrose, 1.34% lactose, 0.24% fructose, and 0.19% glucose), 24.5% from proteins, and 13.1% from fat. The high-fat diet (58Y1 Test Diet) had 20.3% calories from carbohydrates (16.15% maltodextrin, 8.85% sucrose, and 6.46% powdered cellulose), 18.1% from proteins, and 61.6% from fat (31.7% lard and 3.2% soybean oil). In the second phase, healthy control mice were kept with the standard diet (ST-ST10; *n* = 8), and the overweight mice were shifted to the standard diet alone (HF-ST10; *n* = 8) or supplemented with agavins (HF-ST + A10; *n* = 8) or oligofructose (HF-ST + O10; *n* = 8) for five more weeks. Food and water were provided ad libitum throughout the experiment.

Mice experiments were conducted according to the Mexican Norm NOM-062-ZOO-1999 and approved by the Institutional Care and Use of Laboratory Animals Committee from Cinvestav-Mexico (CICUAL; protocol number 0091-14).

### 2.2. Agavins and Oligofructose Fructans

Agavins from four-year-old *Agave tequilana* Weber blue variety plants were extracted and purified in our laboratory and presented an average DP of 8 [21]. Oligofructose was bought from Megafarma^®^ (Mexico City, Mexico) and possess an average DP of 5. Agavins and oligofructose were added in the water at a concentration of 0.38 g/mouse/day [15,22].

### 2.3. gDNA Extraction

Cecal contents were collected before and after the fructans supplementation (at five and 10 weeks, respectively). At the end of first and second experimental phase, mice were anaesthetized with a 60 mg/kg intraperitoneal dose of sodium pentobarbital and the gastrointestinal tract was exposed for cecum removal. Cecal content was snap frozen in liquid nitrogen and stored at −70 °C until their use. Genomic DNA was extracted using the ZR Fungal/Bacterial DNA MiniPrep (Irvine, CA, USA), following the manufacturer’s instructions. The concentration and purity of DNA were evaluated using a Nanodrop spectrophotometer. Extracted DNA was stored at −20 °C until its use.

### 2.4. PCR Amplification of the V4 Region of the Bacterial 16S rRNA Gene

To assess microbial composition, the V4 region of the bacterial 16S *rRNA* gene was amplified with barcoded fusion primers (F515/R806) [23]. PCR reactions were carried out in triplicate, 25 μL reactions with 5 μM forward and reverse primers, 2 μL template DNA, and 1X of HotMasterMix (5 PRIME, Gaithersburg, MD, USA). Thermal cycling of PCR reactions consisted of an initial denaturation at 94 °C for 3 min, followed by 35 cycles of denaturation at 94 °C for 45 s, annealing at 50 °C for 1 min, and extension at 72 °C for 90 s, with a final extension of 10 min at 72 °C.

### 2.5. Amplicon Quantitation, Pooling, and Sequencing

DNA concentration for each amplicon was measured using the Quant-iT PicoGreen dsDNA reagent and kit (Thermo Scientific, Waltham, MA, USA). Assays were carried out using 2 μL of cleaned PCR product in a total reaction volume of 200 μL in black, 96-well microtiter plates. Fluorescence was measured on a BioTek Synergy HT plate reader using the 480/520-nm excitation/emission filter pair. Following quantitation, cleaned amplicons were combined in equimolar ratios into a single tube. The final concentration of the pooled DNA was determined using the Qubit high-sensitivity dsDNA assay (Invitrogen, Carlsbad, CA, USA). Sequencing was carried out on the Illumina MiSeq platform at New York University.

### 2.6. Sequence Analysis

Sequences were processed and analyzed in the QIIME software package (Quantitative Insights Into Microbial Ecology, v1.8.0, La Jolla, CA, USA) following the pipeline described by Caporaso et al. [24]. Sequences were removed from the analysis if they were <200 or >350 nt in length, had a mean quality score < 20, contained ambiguous characters, contained an uncorrectable barcode, or did not contain the primer sequence. Remaining sequences were assigned to samples by examining the 12-nt barcode. Similar sequences were clustered into operational taxonomic units (OTUs) using the open reference method. Taxonomic assignments for each OTU were made using the Greengenes database (May 2013) with a minimum identity of 97%. Finally, an OTU table was used to generate relative abundance plots and to calculate alpha and beta diversity (alpha diversity refers to the diversity within each sample, and beta diversity refers to patterns of similarities and differences among samples). All communities were rarefied up to 6525 reads per sample to calculate the bacterial diversity.

The raw sequences supporting the results of this article are available in the NCBI Sequence Read Archive repository under accession no. SRX1532779.

### 2.7. LEfSe Analysis

Linear discriminant analysis effect size (LEfSe) was used to detect significant changes in relative abundance of microbial taxa between overweight mice fed with the standard diet and fructans supplements. Briefly, LEfSe is an algorithm for applying 16S *rRNA* gene datasets to detect bacterial organisms that are differentially abundant between two or more microbial environments [25]. LEfSe first identifies features that are significantly different among biological classes using the non-parametric factorial Kruskal-Wallis ran-sum test, and then LEfSe utilizes linear discriminant analysis (LDA) to estimate the effect of each differentially-abundant feature.

### 2.8. SCFA and pH Determinations

A weight of 0.05 g of homogenized cecal content was placed in a conic tube. The pH was measured directly in the cecal sample through insertion of a microelectrode (PHR-146, Lazar Research Laboratories Inc., Los Angeles, CA, USA) in the tube. The pH value was read when stability was achieved; after each reading, the microelectrode was removed and rinsed with distilled water. SCFA analysis was carried out in the same sample using gas chromatography and flame ionization detection (GC-FID) [26]. Briefly, 0.3 mL of Milli-Q water was added to the tube with cecal content. The solution was acidified with 0.05 mL of H_2_SO_4_ and SCFA were extracted by shaking with 0.6 mL of diethylether and subsequent centrifugation at 10,000× *g* for 30 s. One microliter of the ether phase was injected directly onto a Nukol^™^ capillary column (30 m × 0.32 mm; Supelco, Bellefonte, PA, USA) using an injector temperature of 180 °C and nitrogen as the carrier gas. The column temperature was initially 80 °C, then increased to 120 °C at 15 °C/min and kept at this temperature for 10 min, following an increment to 200 °C at 10 °C/min and remaining at this temperature for 10 min. The detector temperature was 230 °C. The identification and quantification of the SCFA were carried out using the retention times and calibration curves for each acid, respectively.

### 2.9. Statistical Analysis

Results are presented as mean ± SEM. Differences between ST5 and HF5 groups were assessed by Student’s *t* test. Differences between the diets were determined using a one-way ANOVA followed by Bonferroni’s multiple comparison tests. Differences were considered significant when *p* < 0.05. Statistical analyses were performed using GraphPad Prism (GraphPad Software, La Jolla, CA, USA).

## 3. Results

To assess the impact of agavins on cecal microbiota of overweight mice, we sequenced V4 amplicons of 16S rRNA genes. After trimming, assembly, and quality filtering, we obtained a total of 635,054 sequence reads from 42 samples using a MiSeq sequencing platform. The average sequence read was 15,041 ± 1561 per sample (Supplementary Table S1).

The results of present work showed that the mouse cecal microbiota was greatly dominated by three phyla (Firmicutes, Bacteroidetes, and Proteobacteria) with six other minor phyla (Tenericutes, Actinobacteria, Cyanobacteria, Defferribacteres, Verrucomicrobia, and TM7; Supplementary Figure S2).

### 3.1. High-Fat Diet Induced Overweight and Altered Microbial Diversity and Composition

**Figure 1:**
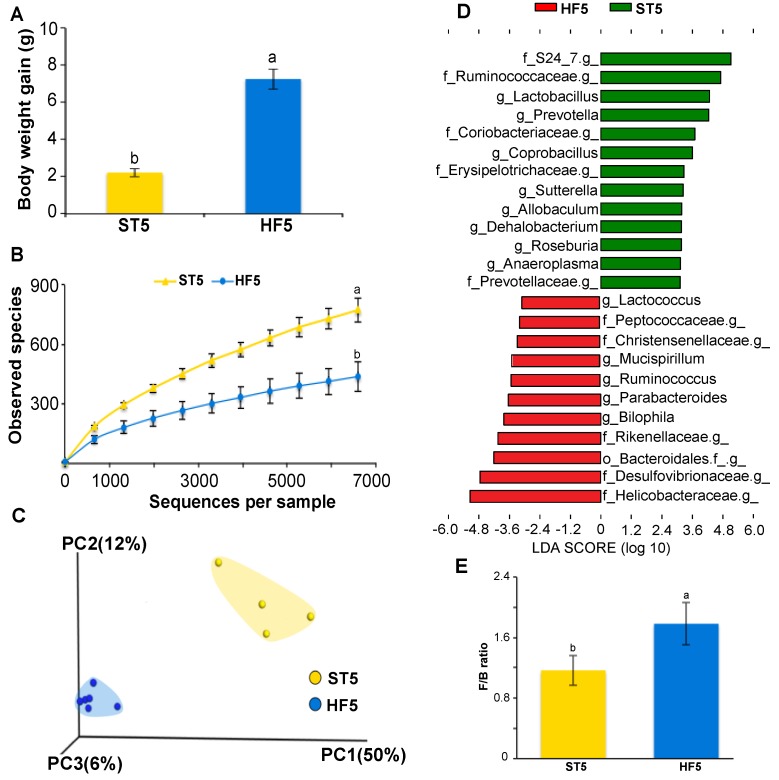
High-fat diet consumption for five weeks induced overweight and modified the cecal microbiota composition of mice. Body weight gain (**A**); bacterial alpha diversity in cecum according to diet (**B**); principal coordinate analysis (PCoA) plot of cecal communities (**C**); linear discriminant analysis showing the differentially-overrepresented genera between mice fed with standard and high-fat diets (**D**); and the effect of the diet on the Firmicutes/Bacteroidetes ratio (**E**). Treatments with different superscript letters indicate significant differences (*t* test, *p* < 0.05).

At the end of the first phase trial, after a high-fat diet consumption for five weeks, HF5 mice showed a significant increase in body gain weight (reaching overweight levels [27]) in relation to the ST5 group (7.26 ± 0.54 g vs. 2.22 ± 0.23 g, respectively; *t* test, *p* < 0.001; Figure 1A); which was associated with a substantial loss of bacterial alpha diversity in cecum of HF5 mice, compared to the standard diet control group ST5 (438 ± 61 vs. 774 ± 75, the number of observed species, respectively; non-parametric *t* test, *p* < 0.05; Figure 1B). In addition, a clear separation of bacterial structures between mice fed with the high-fat or standard diet was observed (weighted UniFrac distances; PERMANOVA, *p* < 0.05; Figure 1C).

On the other hand, HF5 mice were characterized by the increased relative abundance in approximately 41% of Proteobacteria (Helicobacteraceae and Desulfovibrionaceae families, including the *Bilophila* genus) and decreased in about of 17% the Firmicutes (Ruminococcaceae, Lactobacillaceae, Erysipelotrichaceae, Lachnospiraceae, and Dehalobacteriaceae families, including the genera *Lactobacillus*, *Coprobacillus*, *Allobaculum*, *Roseburia*, and *Dehalobacterium*) and approximately 25% the Bacteroidetes (S24_7 and Prevotellaceae families including *Prevotella* genus) (LDA > 3.0; Figure 1D and Supplementary Figure S3), with an increase of the Firmicutes/Bacteroidetes ratio (1.78 vs. 1.17 for HF5 and ST5, respectively; *t* test, *p* < 0.05; Figure 1E).

High-fat diet consumption not only induced alterations in the body weight and composition of the cecal microbiota, but also changed the biochemical environment and microbiota activity in the cecum of HF5 mice which showed a significant reduction of SCFA levels and an increment of pH in the cecal content in relation to the standard diet (ST5) group (*t* test, *p* < 0.05; Table 1).

### 3.2. Diet Shift Induced Body Weight Loss and Restored Altered Microbial Diversity in Overweight Mice

**Figure 2:**
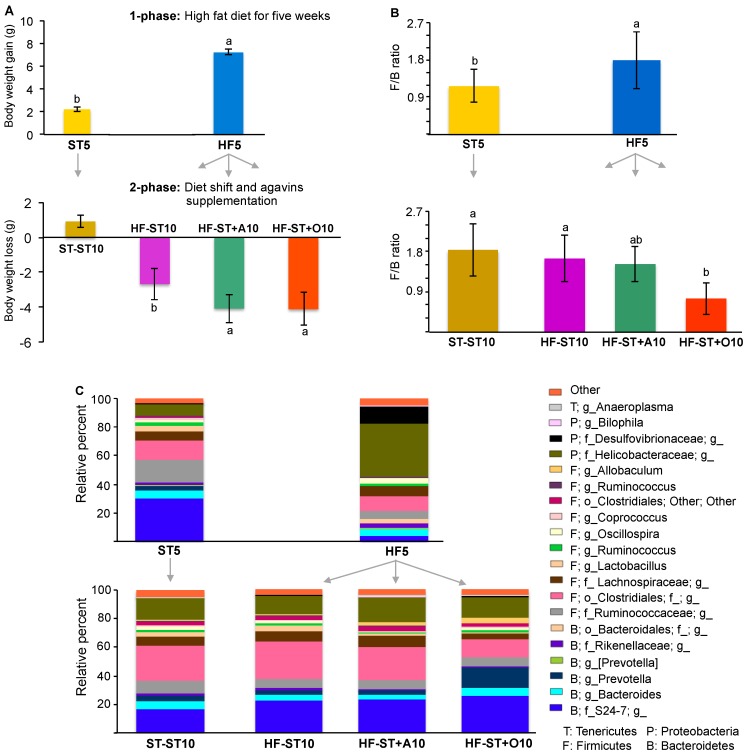
The effect of the diet shift and prebiotic supplementation on body weight loss and cecal microbiota composition in overweight mice. Body weight loss (**A**); Firmicutes/Bacteroidetes (F/B) ratio after the switch to a standard diet alone, or supplemented with agavins or oligofructose (**B**); differences in relative abundance of bacterial taxa in cecum according to diet group (**C**). Each taxon representing >1% of the average relative abundance in study groups is indicated by a different color.

At the end of the second phase, after overweight mice were switched for five weeks to the standard diet, HF-ST10 mice exhibited a body weight loss (Figure 2A). In addition, the return to standard diet in all cases—with or without prebiotic supplementation—decreased the Firmicutes/Bacteroidetes ratio from 1.78, observed in HF5, to 1.64, 1.52, and 0.75 for HF-ST10, HF-ST10 + A, and HF-ST + O, respectively (Bonferroni’s test, *p* < 0.05; Figure 2B), as well as the relative abundance of Proteobacteria in approximately 34% (Helicobacteraceae and Desulfovibrionaceae families; Figure 2C).

**Figure 3:**
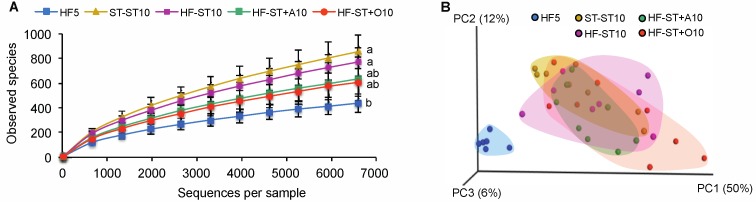
The effect of the diet shift and prebiotic supplementation on the cecal microbiota composition of overweight mice. Bacterial alpha diversity in cecum according to diet group (**A**); treatments with different superscript letters indicate significant differences (Bonferroni’s test, *p* < 0.05). Principal coordinate analysis (PCoA) plot of cecal communities (**B**). Weighted UniFrac distances were used to evaluate beta diversity.

HF-ST10 mice were characterized by a complete restoration of bacterial alpha diversity in relation to the HF5 group (771 ± 115 vs. 438 ± 61, the number of observed species, respectively; non-parametric *t* test, *p* > 0.05; Figure 3A), as well as the bacterial community structures in cecum, compared to the standard diet (ST-ST10) group (weighted UniFrac distances; PERMANOVA, *p* > 0.05; Figure 3B).

**Figure 4:**
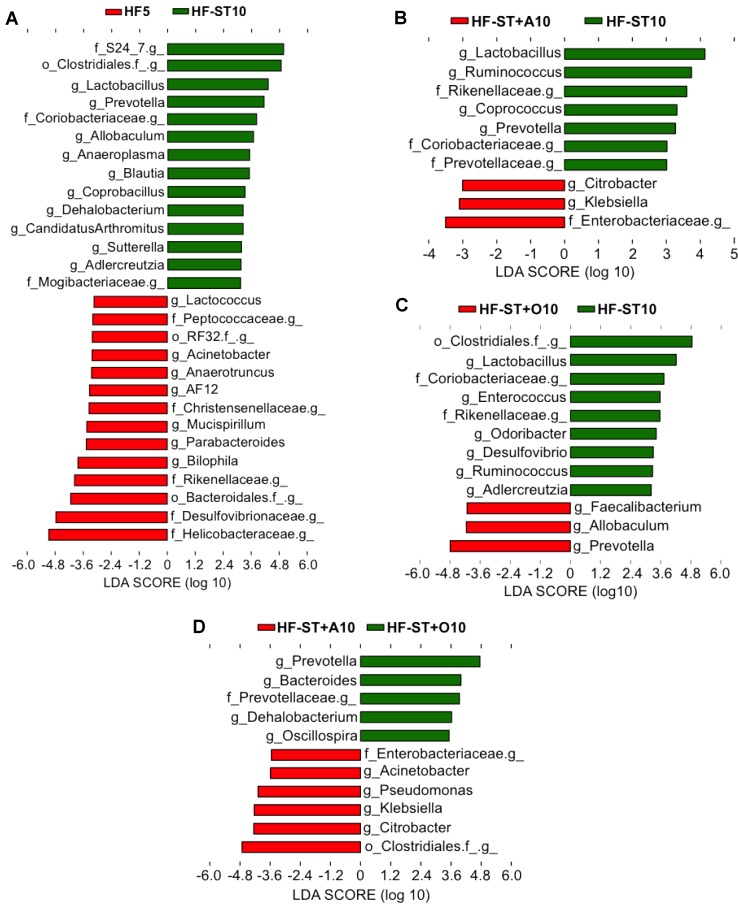
Linear discriminant analysis showing the differentially-overrepresented genera between overweight mice fed with: a high-fat diet and the diet shift (**A**); the diet shift and agavins supplement (**B**); the diet shift and oligofructose supplement (**C**); and agavins and oligofructose supplements (**D**). LDA effect size (3.0-fold) was used to determine the significant biomarkers.

Furthermore, the HF-ST10 group showed a drastic change on the cecal microbiota composition in relation to the HF5 group, increasing the abundance of the genera *Lactobacillus*, *Prevotella*, *Allobaculum*, *Anaeroplasma*, *Blautia*, *Coprobacillus*, *Dehalobacterium*, *Candidatus Arthromitus*, *Sutterella*, and *Adlercreutzia* and decreased *Lactococcus*, *Acinetobacter*, *Anaerotruncus*, *AF12*, *Mucispirillum*, *Parabacteroides*, and *Bilophila* (LDA > 3.0; Figure 2C and Figure 4A).

Moreover, the diet shift led HF-ST10 mice to recover the biochemical environment and microbiota activity in the cecum, displaying similar SCFA concentration and pH values in relation to the standard diet (ST-ST10) group (*t* test, *p* > 0.05; Table 2).

### 3.3. Effects of Prebiotic Supplementation on Cecal Microbiota in Overweight Mice

In contrast, to standard diet alone, overweight mice that were shifted to the standard diet and received any prebiotic treatments (agavins or oligofructose) exhibited an accelerated body weight loss (Figure 2A), as well as a partial restoration of the cecal diversity in relation to the HF5 group (635 ± 177 and 607 ± 117, the number of observed species for HF-ST + A and HF-ST + O, respectively, vs. 438 ± 75, the number of observed species; non-parametric *t* test, *p* < 0.05; Figure 3A). Alpha bacterial diversity was not significantly different between mice fed agavins (branched fructans) or oligofructose (linear fructans). Interestingly, bacterial community structures from the agavins and oligofructose supplementation were not different to those from the unsupplemented standard diet (non-parametric *t* test, *p* < 0.001; Figure 3B and Supplementary Figure S4A). Noticeably, the HF-ST + O10 group showed the highest dispersion along the PC1 axis in relation to HF-ST + A10 and ST-ST10 groups (non-parametric *t* test, *p* < 0.01; Figure 3B and Supplementary Figure S4B). In addition, the cecal microbiota of the agavins-supplemented diet group (HF-ST + A10) was more similar to the standard diet groups (ST-ST10 or HF-ST10) compared to the oligofructose (HF-ST + O10) (weighted UniFrac distance; *t* test, *p* < 0.05; Supplementary Figure S4C,D).

On the other hand, the supplementation of agavins (HF-ST + A10) or oligofructose (HF-ST + O10) was associated with different communities: in relation to HF-ST10 group, agavins increased two genera (*Citrobacter* and *Klebsiella*) and decreased four genera (*Lactobacillus*, *Ruminococcus*, *Prevotella*, and *Coprococcus*), while oligofructose increased three genera (*Prevotella*, *Faecalibacterium* and *Allobaculum*) and decreased six genera (*Lactobacillus*, *Enterococcus*, *Odoribacter*, *Adlercreutzia*, *Desulfovibrio*, and *Ruminococcus*). In relation to oligofructose, agavins increased *Citrobacter*, *Klebsiella*, *Pseudomonas*, and *Acinetobacter*, and decreased *Prevotella*, *Bacteroides*, *Dehalobacterium*, and *Oscillospira* (LDA > 3.0; Figure 2C and Figure 4B–D).

In the same way as the diet shift alone, prebiotic (agavins or oligofructose) supplementation modified not only the cecal microbiota composition, but also the microbiota activity. However, supplementation with agavins (HF-ST + A10) or oligofructose (HF-ST + O10) significantly increased the concentration of acetic, propionic, and butyric acids with a noticeable reduction of pH in the cecal content in relation to non-supplemented controls (ST-ST10 and HF-ST10; Bonferroni’s test, *p* < 0.1; Table 2).

## 4. Discussion

We previously reported that agavins supplementation to a standard diet reverted the metabolic syndrome (including body weight loss) induced by high-fat diet consumption [21]. However, we do not know that the changes originated with agavins consumption on the gut microbiota, which could be associated with this effect. Therefore, the present study describes the changes in the cecal microbiota, SCFA production, and pH values associated with body weight loss in overweight mice.

High-fat diet consumption for five weeks significantly increased the body weight gain of mice, and also led to a substantial decrease of bacterial diversity in the cecal microbiota (Figure 2A and Figure 3A); which is consistent with the effects of fat in reducing diversity, as previously reported [28,29,30].

Firmicutes and Bacteroidetes are the most abundant members of the cecal microbiota, however, the ratio of these bacterial groups can change over time or by different factors, such environment and diet (especially those with a high fat content) [18,19,20]. An increase of the ratio of Firmicutes/Bacteroidetes was seen in overweight mice, which has been associated with obesity [18,20].

We found that a high-fat diet enriched *Bilophila*, a genus that include some opportunistic pathogens (for example *B. wadsworthia* [31]). Microbial changes under a high fat diet reduced cecal SCFA concentrations and increased pH, which is consistent with altered microbial metabolic activity, as previously reported [32].

Supplementation with agavins or oligofructose showed an accelerated body weight loss with partially restored the cecal microbiota diversity (Figure 2A and Figure 3A), as well as an increase in the SCFA concentrations and acidic pH (Table 2). This might be mediated by selected supplement addition for specific bacterial taxa that tolerate a more acidic pH [33], since the direct effect of probiotic supplements on the microbiota have not been demonstrated [9,14,34]. Moreover, acetic acid suppresses appetite [35] and propionate and butyrate acids modulate hormones, such as GLP-1 and PYY, involved in satiety [5,6,7], and this mechanism might also contribute to body weight loss.

Weight loss was associated with a decrease in the Firmicutes/Bacteroidetes ratio, as in previous reports [18,36], and the effect is due, in part, to a reduction of caloric intake in fructans-supplemented mice [21]. Taxa associated with greater body weight loss included *Klebsiella* and *Citrobacter* (Enterobacteriaceae; Figure 4B). Enterobacteriaceae have also been reported to increase during weight loss in obese mice [37] and humans [38,39]. Other bacteria enriched by supplementation with oligofructose included *Prevotella*, *Allobaculum*, and *Faecalibacterium* genera (Figure 4C). Similarly, a previous study has reported an association between *Allobaculum* and a reduction of body weight in obese mice [17].

Interestingly, agavins and oligofructose supplementation led to the highest cecum SCFA, despite of structural differences between these fructans. However, fructan structure and the degree of polymerization were associated with differences in the bacteria genera enriched by agavins (branched) or oligofructose (linear). In relation to supplementation with oligofructose, agavins supplementation enriched *Citrobacter*, *Klebsiella*, *Pseudomonas*, and *Acinetobacter*, and decreased *Prevotella*, *Bacteroides*, *Dehalobacterium*, and *Oscillospira* (Figure 4D). Nevertheless, both supplements shared the physiological response of accelerating body weight loss perhaps due to functional redundancy of the gut microbiota [40].

## 5. Conclusions

In conclusion, diet supplementation of agavins restored microbiota diversity depleted by a high-fat diet, reduced the Firmicutes/Bacteroidetes ratio, enriched members of the Enterobacteriaceae, and increased the SCFA concentration in cecum, which could induce an accelerated weight loss in mice. These results could provide novel insight to develop a new supplementary strategy using agavins to modulate gut microbiota in overweight or obese individuals, which might have positive consequences on body weight loss. Furthermore, the enrichment of members of Enterobacteriaceae has not been reported previously under a prebiotic supplement, which opens opportunities to explore new probiotics.

## Figures and Tables

**Table 1:** The effect of high-fat diet intake for five weeks on short-chain fatty acid concentration and pH in the cecal content of mice.

Group	Acetic Acid *	Propionic Acid *	Butyric Acid *	pH
ST5	15.13 ± 0.92 ^a^	3.85 ± 0.20 ^a^	4.54 ± 0.33 ^a^	7.60 ± 0.12 ^b^
HF5	5.43 ± 0.65 ^b^	1.96 ± 0.11 ^b^	1.72 ± 0.09 ^b^	8.18 ± 0.06 ^a^

ST5: mice fed with a standard diet; HF5: mice fed with a high-fat diet. Data are shown as mean ± SEM. Means with different letters (a,b) indicate significant differences (*t* test, *p* < 0.05). * µmoles/g of wet weight.

**Table 2:** The effect of the diet shift and agavins supplementation on short-chain fatty acid concentration and pH in the cecal content of overweight mice.

Group	Acetic Acid *	Propionic Acid *	Butyric Acid *	pH
ST-ST10	20.73 ± 2.07 ^c^	6.07 ± 0.60 ^a,b^	6.53 ± 0.60 ^a,b^	7.65 ± 0.07 ^a^
HF-ST10	26.68 ± 0.69 ^b^	6.48 ± 0.26 ^b^	6.16 ± 0.39 ^b^	7.26 ± 0.08 ^a^
HF-ST + A10	34.27 ± 1.77 ^a^	7.16 ± 0.44 ^a^	7.51 ± 0.46 ^a^	6.92 ± 0.03 ^b^
HF-ST + O10	34.89 ± 1.85 ^a^	7.73 ± 0.29 ^a^	6.95 ± 0.40 ^a^	6.79 ± 0.06 ^a^

ST-ST10: healthy mice fed with the standard diet for ten weeks. HF-ST10: overweight mice switched for five weeks to the standard diet alone; or supplemented with agavins (HF-ST + A10) or oligofructose (HF-ST + O10). Data are shown as mean ± SEM. Means with different letters (a,b,c) indicate significant differences (Bonferroni’s test, *p* < 0.1). * µmoles/g of wet weight.

## Supplementary Materials

The following are available online at www.mdpi.com/2072-6643/9/9/821/s1, Figure S1: Experimental design, Figure S2: Relative average abundance of bacterial phyla in the cecal microbiota of mice by diet, Figure S3: Differences in relative abundance of bacterial taxa in cecum between mice fed with a high-fat diet or standard diet for five weeks, Figure S4: Weighted UniFrac distances according to diet group, Table S1: Sequencing yield and operational taxonomic units obtained through MiSeq sequencing analysis.
