# Supplementary material for: Modulation of Gut Microbiota of Overweight Mice by Agavins and Their Association with Body Weight Loss
Source: Nutrients. 2017 Aug 23;9(9):821. doi: 10.3390/nu9090821 (PMC5622678; doi:10.3390/nu9090821)
Supplement: Supplementary file 1 [file nutrients-09-00821-s001.pdf]

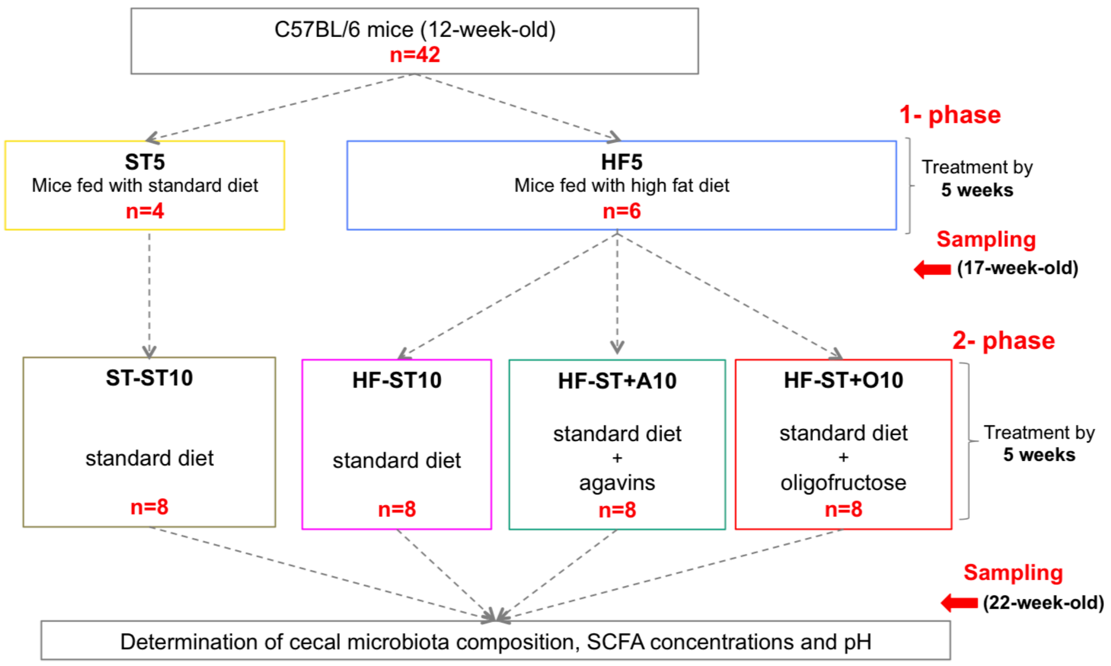

Supplementary Figure S1. Experimental design. Initially, forty-two male C57BL/6 mice (12 weeks old) were randomly divided into two groups. Mice were subject to 2-phase trial, the first one to gain weight and the second one to lose weight. In the first phase mice were fed with standard (n=12; 5053 Lab Diet) or high fat diet (n=30; 58Y1 Test Diet) for 5 weeks. In the second phase, healthy control mice were kept with the standard diet (ST-ST10; n=8), and the overweight mice were shifted to the standard diet alone (HF-ST10; n=8) or supplemented with agavins (HF-ST+A10; n=8) or oligofructose (HF-ST+O10; n=8) for 5 more weeks. Body weight was registered and cecal contents were collected at 5 and 10 weeks (before and after of prebiotics supplementation, respectively) for analysis of the cecal microbiota composition, SCFA concentrations and pH values.

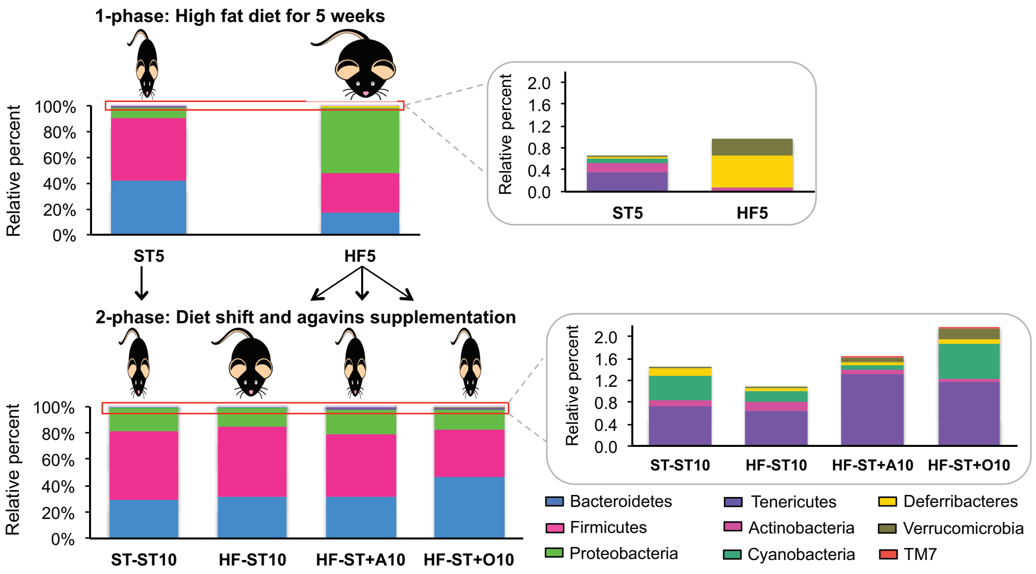

Supplementary Figure S2. Relative average abundance of bacterial phyla in the cecal microbiota of mice by diet at the end of the first and second phase trial.

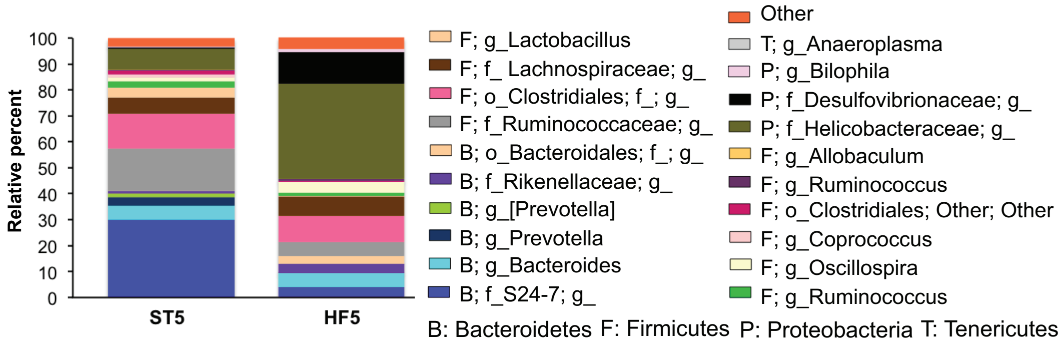

Supplementary Figure S3. Differences in relative abundance of bacterial taxa in cecum between mice fed with high fat diet or standard diet for five weeks. Each taxon representing >1% of the average relative abundance in study groups is indicated by a different color.

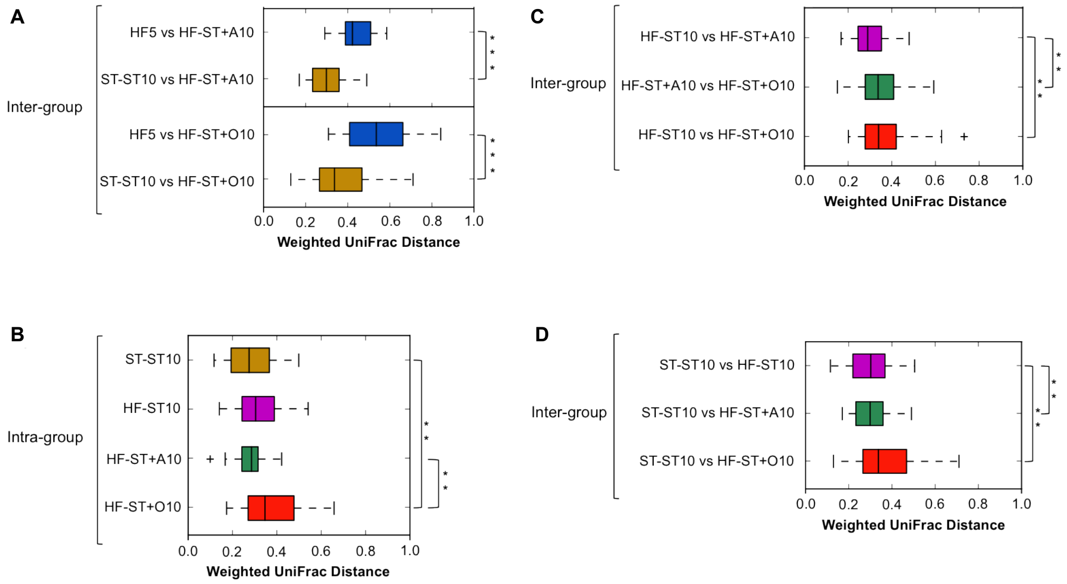

Supplementary Figure S4. Weighted UniFrac distances according to diet group. Box plots of inter-group distances of bacterial communities between the overweight mice (HF5) or healthy control mice (ST-ST10) and overweight mice that were shifted to the standard diet with agavins (HF-ST+A10) or oligofructose (HF-ST+O10) supplement (*** *P* < 0.001, non-parametric t-test) (**A**); Box plots of intra-group distances of bacterial communities between the control mice (ST-ST10) and overweight mice that were shifted to the standard diet alone (HF-ST10) or supplemented with agavins (HF-ST+A10) or oligofructose (HF-ST+O10; ***P* < 0.01, non-parametric t-test) (**B**); Box plots of inter-group distances of bacterial communities between the overweight mice that were shifted to the standard diet alone (HF-ST10) or supplemented with agavins (HF-ST+A10) or oligofructose (HF-ST+O10; ***P* < 0.05, non-parametric t-test) (**C**); Box plots of inter-group distances of bacterial communities between healthy control mice (ST-ST10) and the overweight mice that were shifted to the standard diet alone (HF-ST10) or supplemented with agavins (HF-ST+A10) or oligofructose (HF-ST+O10; ***P* < 0.01, non-parametric t-test) (**D**).
